# Pattern of Animal Bites and Delays in Initiating Rabies Postexposure Prophylaxis among Clients Receiving Care in Korle-Bu Teaching Hospital

**DOI:** 10.1155/2020/6419104

**Published:** 2020-05-26

**Authors:** Joyce A. Addai, Benjamin D. Nuertey

**Affiliations:** ^1^Medical Out Patient Department, Korle-Bu Teaching Hospital, Accra, Ghana; ^2^Community Health Department, University of Ghana Medical School, Accra, Ghana; ^3^Public Health Department, Tamale Teaching Hospital, Tamale, Ghana

## Abstract

**Introduction:**

Each year, an estimated 25000 rabies death occurs in Africa. Post-exposure prophylaxis (PEP) remains the only proven approach in preventing rabies deaths. Most of rabies deaths occur among those who delayed, did not receive, or complete rabies PEP. The aim of this study was to determine the pattern of animal bite, factors associated with delays in initiating, and nonadherence to rabies PEP regimen.

**Method:**

Data on clients reporting for rabies PEP in a tertiary hospital setting in Ghana were collected from 2013 to 2016. Demographics, place, and source of exposure were collected. Other information obtained included adherence to the PEP protocol and GPS coordinates of the town of animal bites. The shortest distance between the GPS coordinates of town of animal bite and the site of administration of the rabies PEP was calculated. A total of 1030 individuals received rabies PEP over the period.

**Results:**

Overall, 52.9% (545) were males while 47.1% (485) were females. Majority of the cases were between the age group 1–10 years accounting for 29.2%. Dog bites accounted for 96.5% (*n* = 994). Cats, nonhuman primates, human bites, respectively, accounted for 1.8% (*n* = 18), 1.2% (*n* = 12), and 0.6% (*n* = 6) of all bites. Majority of bites occurred at home (66.2% (*n* = 646)). Also, 31.6% (*n*  = 308) and 2.3% (*n* = 22) of bites occurred on the streets and neighbors/friends' homes, respectively. Only, 18.7% initiated PEP within 24 hours of bite. Rabies PEP regimen completion rate was 95.2% (*n* = 976). The median distance travelled to access rabies PEP was 7.87 km (IQR, 3.58–16.27) km. Overall, 34.7% (*n* = 344) had the animal bite within 4.99 km radius of the treatment room of KBTH. Clients who delayed in initiating rabies PEP were 2.6 (95% CI, 1.3–5.1) times more likely to be males and 2.0 (1.1–3.9) times more likely to receive bites in a location more than 5 km radius from the KBTH treatment room. Gender, age, and distance of bite from the treatment room were not associated with the likelihood of not completing rabies PEP schedule. *Discussion*. Bites from suspected rabies infected animals remain a problem in southern Ghana. There are significant delays in initiating PEP for rabies prevention. Most animal bite victims are children ten years and below. Male gender and bites more than 5 km radius from the site of rabies PEP administration were most significant factors associated with delays in initiating rabies PEP. There is the need for adopting strategies to encourage prompt initiation and adherence to PEP.

## 1. Introduction

Rabies is a fatal infection caused by a virus that can affect almost all mammals. However, dogs accounts for about 99% of human rabies cases [1]. Worldwide death from rabies is estimated at about 59,000 annually [2, 3]. A significant proportion of rabies deaths occur in Africa. Each year, 44% (*n* = 25000) of worldwide rabies death occurs in Africa [4]. In Ghana, the incidence of rabies has been on the increase [5, 6]. Rabies infection is associated with extreme suffering characterized by convulsions, violent muscular spasm, aggression, hydro and photophobia [7]. Most of the victims of rabies are children [7, 8]. The World Health Organization has announced the global goal of eliminating rabies death by the year 2030; however, with the pace of development, this target may not be met if drastic measures are not taken [9, 10].

Rabies has an almost worldwide distribution, currently present in about 100 countries in all continents except Antarctica and Australia [11]. The rabies virus belongs to the genera Lyssavirus and the family Rhabdoviridae [12]. Bites from rabid animals are the significant route of transmission of rabies. The incubation period of rabies is highly variable ranging from 2 weeks to 6 years; and the average incubation period however is within 2-3 months [13]. Greater risk for rabies bites are bites on the hands, neck, and face because of shorter length and increased number of neurons within such areas [13]. Rabies still poses a significant public health challenge throughout Africa. In Africa, the best approach in preventing human rabies has been attributed to dog rabies parenteral vaccination [14]. However, most dogs are not vaccinated due to weak legislative and enforcement issues prevalent in most parts of Africa. Laboratory confirmation of suspected rabies cases in rabies endemic countries is rarely carried out due to financial constraints, lack of requisite training, and resources and even when available, takes too long a time to receive results making it impractical for clinical use [15]. With all this challenges, postexposure prophylaxis remains the most efficient approach at preventing human rabies deaths postexposure. It is estimated that about 15 million receives rabies PEP annually [16]. Most often, rabies PEP is not available in rural areas where it is most needed [3]. Some clients often report late due to long travel distance from site of bite to center for rabies PEP, which constitute significant delays in initiating rabies PEP. Studies have found significant delays in the initialization of rabies PEP [17] which affect the efficacy of rabies PEP. Delays in rabies PEP initiation affect the overall quality of the efficacy of the PEP. However, some rabies exposed individuals in Ghana initiate rabies PEP late and others do not complete the schedules for rabies PEP. For the purpose of better counseling for clients reporting for rabies PEP, it is essential to undertake this review and find out-patient characteristics that predispose to noncompliance to rabies PEP schedule so as to intensify adherence counseling for such individuals.

The treatment room of Korle-Bu teaching hospital's (KBTH) medical out-patient department (OPD) is the center for rabies PEP in Korle-Bu teaching hospital attending to about 350 rabies exposed clients annually. Over the past three years, over 1000 rabies exposed individuals received rabies postexposure prophylaxis. The aim of this study was to determine the pattern of animal bites presenting for rabies PEP, patient characteristics, and factors, which predispose to noncompliance to rabies PEP schedules.

## 2. Materials and Methods

### 2.1. Study Design

This is a retrospective study that reviewed service data generated as part of rabies postexposure prophylaxis. Data on animal bite; type of animal, date of bite, place, and demographic characteristics of the person were captured routinely as part of service data. This study reviews the data generated so as to obtain useful information that might influence patient counseling process within the treatment room and in Ghana. The available data, which spanned a three-year period, from January 2013 to December 2016, was reviewed and analyzed.

### 2.2. Study Sites

The study took place in the treatment room of the central out-patient department. Most animal bites client within Accra are referred to the treatment room of Korle-Bu Teaching Hospital (KBTH), Medical out-patient Department (OPD) for postexposure prophylaxis. The treatment room currently has about six nurses and carries out other functions such as vaccination of other antigens, out-patient based wound dressing and injections. Other activities that take place in the treatment room include counseling.

### 2.3. Study Population and Sample Size

The study population for this data review included all patients that took rabies postexposure prophylaxis in the treatment room of the central OPD of KBTH. All clients that took PEP from January 2013 to December 2016 were eligible to be included in the study.

This study aimed at a complete enumeration and review of all data captured within the period under review. About 1030 clients' records were available over the three-year period (2013–2016). All clients that used the service over the period under review for which the data was available and eligible were included in the study.

### 2.4. Data Collection Methods

The already available data were entered in to the EPIDATA and exported in to the STATA for analysis. Personal identifiers such as names were not entered save serial numbers generated as part of the rabies PEP administration. Ages, sex, place of bite, type of animal bite, date of bite among others were entered for each client. The coordinates (longitude and latitude) of town of aggression were obtained by entering the place of bite into Google Maps. The coordinates were obtained one by one. All addresses were geo-referenced.

### 2.5. Statistical Analysis

Descriptive statistics were obtained for most variables. This included the percentage of bites that occurred at home, street, or neighbor's house. We also obtained percentages for types of bites. GPS coordinates of town of bite were obtained by entering the place of bite into mapping software online, Google Map. The approximate straight-line distance in kilometers from the place of bite to the treatment room was determined using STATA. The distances were grouped for analysis. Inferential statistics displayed odds ratio as measure for association in a logistic regression of factors associated with delay in initiation rabies PEP.

### 2.6. Ethical Issues

Ethical clearance was obtained from the institutional ethical review board of Korle-Bu teaching hospital. Permission to carry out the study was granted by the scientific and technical committee of Korle-Bu teaching hospital. There were no direct patient contacts in the entire process of this retrospective review. Confidentiality was maintained. Data were password-protected. Patient identifier such as name of patient was not entered during the data entry process. The study followed the data protection guidelines of KBTH.

## 3. Results

### 3.1. Background Characteristics

**Figure 1:**
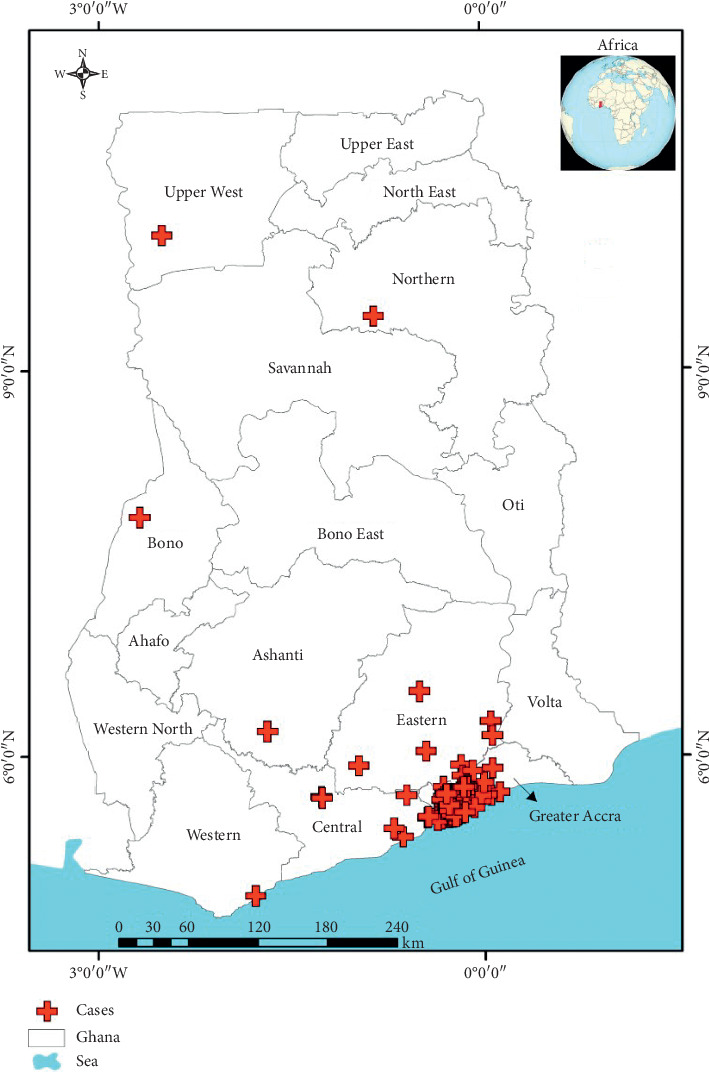
Town/cities of cases reporting at the treatment room of KBTH in Ghana.

**Figure 2:**
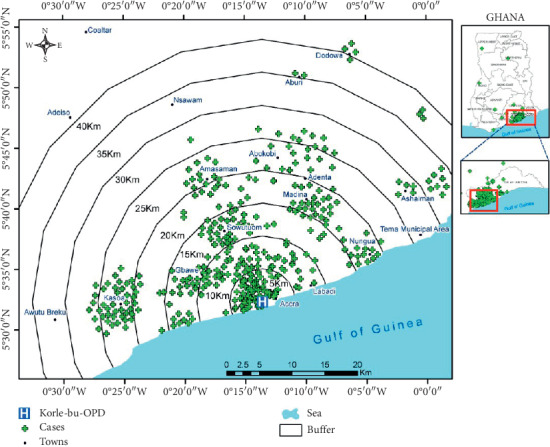
Spot map showing distribution of animal bites reporting at the treatment room of KBTH.

**Figure 3:**
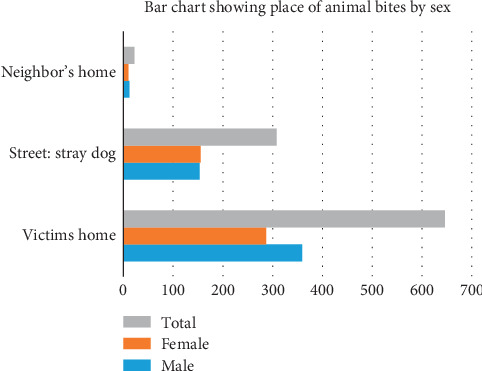
Bar chart showing place of animal bite by sex.

A total of 1030 participants were included in this study.  Table 1 displays the background characteristics of all participants. In all, 52.9% (545) were males while 47.1% (485) were females. The median age was 22 years (interquartile range = 27 years). Majority of suspected rabies exposed receiving rabies postexposure prophylaxis were between the age group 1–10 years accounting for 29.2% (*n* = 290). Also, 18.7% of suspected rabies exposed individuals were between the age group 10 to 19 years. More than 65% of all victims of animal bites requiring rabies PEP were below the age 30. The incidence of reported animal bite requiring rabies PEP decreases with increasing age. The elderly population, >59 years old accounted for 5.6% of all suspected rabies exposed presenting in the KBTH over the period. With regards to the type of animal bites: Dog bites accounted for 96.5% (*n* = 994) of all cases of animal bites. Cats, nonhuman primates, human bites, respectively, accounted for 1.8% (*n* = 18), 1.2% (*n* = 12), and 0.6% (*n* = 6) of all bites. Majority of bites occurred at home accounting for 66.2% (*n* = 646). Also, 31.6% (*n* = 308) and 2.3% (*n* = 22) of bites occurred on the streets and neighbors/friends' homes, respectively. This implies that as much as 66.2% of all cases of animal bites are due to animals living in the home of the victims. With regards to time of initiation of rabies PEP, 18.7% initiated PEP within 24 hours of bite. Also, 37.8% and 31.7% initiated PEP within 1-2 days and 3–7 days, respectively. In all, 11.7% initiated treatment after one week. With regards to compliance to PEP schedules, 95.2% (*n* = 976) completed all five doses of intramuscular postexposure prophylaxis. In the southern sector of Ghana, majority of dog bites occurs from November through to March. The median distance travelled to access rabies PEP was 7.87 km (IQR, 3.58–16.27km). Also, 34.7% (*n* = 344) had the animal bite within 4.99 km radius of the treatment room of KBTH. Clients initiating rabies PEP within 24 hours travelled a median 4.27 km (IQR, 2.02–9.66 km). Figures 1 and 2 displays the towns/cities of bites in Ghana and a spot map of case distribution in and around Accra, respectively.  Figure 3 displays place of animal bites by sex.

Table 2 displays independent factors associated with delays in seeking Rabies PEP. Males were 2.6 (95% CI, 1.3–5.1) times more likely to delay in initiating rabies PEP. Clients who delayed initiating rabies PEP were 2.0 (1.1–3.9) times more likely to receive their respective bites more than 5 km radius away from the KBTH treatment room. Gender, age, and distance of bite from the treatment room was not associated with the likelihood of completing rabies PEP schedule.

## 4. Discussions

The study adds to the evidence that animal bites are important public health concern in Ghana [18]. One significant finding is the higher proportion of animal bites among children compared to adults. Children are most affected in terms of rabies exposure [19, 20] because they are more likely to approach animals without caution [9]. Also, children can be curious and may have inadequate understanding of dog's behavior [21]. Most children bites are however provoked. It is known that adults however are the most affected unprovoked animal bites. A study found out that children were six times more likely to be bitten by a family dog [22]. As high as 66.2% of all cases of animal bites are due to animals living in the home of the victims. This opens a good window for prevention of rabies deaths in Ghana. Efforts to enforce immunization of dogs at home would reduce up to 66% of exposure of rabies, which may be a significant step in preventing rabies infection.

The incidence of reported animal bite requiring rabies PEP decreases with increasing age. Children are likely to sustain more bites compared to older adults due to the fact that children are more likely to provoke an attack. However, the role of adults' health seeking behavior and the tendency to ignore what they term as less significant threats may also play a role in the observed pattern.

Majority of animal bites observed over the period were due to dog bites. The observed pattern may be due to dogs being the predominant pet in the country. Also, exposure to wild animals like jackals and hyenas as observed in other countries [23] were rare in Ghanaian setting. It could be attributed to the fact that the study was conducted in a more urban area situated in the capital of Ghana and as such exposure to such animals were rare. Delays in initiating PEP were rampant with 11.7% initiating PEP after a week. Reasons for delay in other studies cited unaffordability of rabies vaccine [24] and the type of animal bite [25]. However, it is known that, the safest approach in preventing human rabies is to initiate PEP immediately. It has been estimated that in the absence of PEP, 19% of victims bitten by rabid dogs develop rabies [1]. With the high exposure rate to suspected rabies animals, public health actions employing the one-health concept are urgently required to ensure that, Ghana eliminates death from rabies.

The main limitation of the study has to do with self-reported nature of the data generated, which misses community cases of suspected rabies exposure who refused to seek medical care. Some studies have found under reporting of dog bites and suspecting rabies cases at public health facilities in Accra, Ghana [26]. This has the potential of influencing the pattern of animal bites. Also, the study did not follow up clients to determine the incidence of rabies after prophylaxis. Further studies could be directed in that direction. We were unable to collect data on all facilities that the patient visited before visiting the treatment room of the Korle-Bu teaching hospital. This information could explain some of the delays in initiating postexposure prophylaxis.

## 5. Conclusion

In conclusion, animal bites from suspected rabies infected animals are a public health concern in southern Ghana, which require urgent attention to prevent human rabies. There is the need to adequately counsel patients reporting for rabies PEP so as to improve compliance. Since majority of animal bites victims are children, public education by the school health team must include education on how to avoid animal bites among children. Also, most of the animal bites were from animals from victims own home. This provides a window of opportunity to enforce vaccination of all domestic animals, which can serve as a potential reservoir for rabies so as to better prevent human rabies.

## Figures and Tables

**Table 1:** Background characteristics of study participants.

Characteristics	Male	Female	Total	
	*n* (%)	*n* (%)	Number	%
All study participants (*n* = 1,030)	52.9 (545)	47.1 (485)	1,030	
*Age (years) (n* *=* *993)*				
1–9	176 (60.7)	114 (39.3)	290	29.2
10–19	101 (54.3)	85 (45.7)	186	18.7
20–29	83 (46.6)	95 (53.4)	178	17.9
30–39	71 (50.7)	69 (49.3)	140	14.1
40–49	43 (53.4)	39 (47.6)	82	8.3
50–59	29 (47.5)	32 (52.5)	61	6.1
Above 59	27 (48.2)	29 (51.8)	56	5.6
*Type of suspected rabid animal (n* *=* *1030)*				
Dog	531 (53.4)	463(46.6)	994	96.5
Cat	7 (38.9)	11 (61.1)	18	1.8
Nonhuman primates	5 (41.7)	7 (58.3)	12	1.2
Others	2 (33.3)	4(66.7)	6	0.6
*Place of bite (n* *=* *976)*				
Victims home	359 (55.6)	287 (44.4)	646	66.2
Street: stray dog	153 (49.7)	155 (50.3)	308	31.6
Neighbor's home	12 (54.6)	10 (45.5)	22	2.3
*Completion of PEP Schedule (n* *=* *1030)*				
Completed	514 (52.7)	462 (47.3)	976	94.8
Did not complete	30 (61.2)	19 (46.9)	54	5.2
*Average distance travelled from site of bite to PEP Centre (n* *=* *992)*				
Less than 5.00 km	176(51.2)	168(48.8)	344	34.7
5.00–9.99 km	112(49.1)	116(50.9)	228	23.0
10.00–14.99 km	91(53.5)	79(46.5)	170	17.1
15.00 km and above	147(53.0)	103(41.2)	250	25.2

**Table 2:** Independent factors associated with delays in initiating rabies postexposure prophylaxis, adjusting for age and type of bite.

Independent factors	Crude OR	Adjusted OR	
	(95% CI)	Odds ratio	95% CI
*Sex*			
Females		—	
Males	2.6 (1.3–5.1)	2.6	1.3–5.2
*Distance of site of bite from PEP administration*			
Less than 5 km			
More than 5 km	2.0 (1.0–3.9)	2.0	1.1–4.0

## Data Availability

The data are available upon request from the corresponding author.
